# Do Nonalcoholic Fatty Liver Disease and Fetuin-A Play Different Roles in Symptomatic Coronary Artery Disease and Peripheral Arterial Disease?

**DOI:** 10.3390/diseases6010017

**Published:** 2018-02-16

**Authors:** Fabio Nascimbeni, Dante Romagnoli, Stefano Ballestri, Enrica Baldelli, Simonetta Lugari, Valentina Sirotti, Valentina Giampaoli, Amedeo Lonardo

**Affiliations:** 1 Division of Internal Medicine, Ospedale Civile di Baggiovara, Azienda Ospedaliero-Universitaria di Modena–Department of Biomedical, Metabolic and Neural Sciences, University of Modena and Reggio Emilia, Modena 41126, Italy; romagnoli.dante@policlinico.mo.it (D.R.); enrica.baldelli@unimore.it (E.B.); simo_1987@libero.it (S.L.); valentina.sirotti@gmail.com (V.S.); valentina.giampaoli@gmail.com (V.G.); a.lonardo@libero.it (A.L.); 2 Division of Internal Medicine, Hospital of Pavullo–Department of Internal Medicine, Azienda USL, Pavullo, Modena 41126, Italy; stefanoballestri@tiscali.it

**Keywords:** atherosclerosis, coronary artery disease, fetuin-A, nonalcoholic fatty liver disease, peripheral arterial disease

## Abstract

Background and Aims: Nonalcoholic fatty liver disease (NAFLD) is strongly associated with both atherosclerotic cardiovascular disease (CVD) and Fetuin-A. However, the association of Fetuin-A with atherosclerosis is more controversial. We hypothesized that the pathogenic interplay of NAFLD, Fetuin-A and atherosclerosis varies based on arterial site. Accordingly, we aimed to assess NAFLD prevalence, Fetuin-A values and their relationship with symptomatic atherosclerosis occurring in different localizations: coronary artery disease (CAD) vs. peripheral arterial disease (PAD). Methods: One hundred and forty-nine consecutive patients with symptomatic atherosclerotic CVD were recruited: 45 with CAD diagnosed by coronary angiography and 104 with PAD detected by doppler-ultrasound and/or computed tomography angiography and/or angiography. NAFLD was diagnosed based on both ultrasonography and exclusion of competing etiologies. Serum Fetuin-A was measured with ELISA. Results: NAFLD was detected in 54% of the overall group, with higher rates in PAD (59%) than CAD (42%) patients. Median Fetuin-A values were 256 (111–662) μg/mL, higher in patients with CAD (378 (124−662) μg/mL) than those with PAD (236 (111−461) μg/mL). The main findings were: (1) CAD patients had higher Fetuin-A values and less frequently NAFLD than PAD patients; (2) NAFLD was positively associated with Fetuin-A values; however, this association was limited to CAD patients only; (3) Fetuin-A values were positively associated with both CAD and NAFLD. Conclusion: The pathogenic interplay of NAFLD, Fetuin-A and atherosclerosis probably varies according to the arterial site.

## 1. Introduction

Patients with nonalcoholic fatty liver disease (NAFLD) are prone to developing atherosclerotic cardiovascular disease (CVD) independently of classical cardiovascular risk factors, and CVD is a leading cause of mortality in NAFLD [1,2,3]. NAFLD may play a major pro-atherogenic role through a variety of mechanisms [1,2,3,4]. The hepatokine Fetuin-A is gaining attention on the grounds that Fetuin-A mRNA expression in hepatocytes and serum Fetuin-A levels are increased in those with liver steatosis [5,6,7]. Biologically, Fetuin-A serves a dual function: on the one hand, it is a powerful inhibitor of vascular ectopic calcification [8]; on the other hand, it is associated with insulin resistance (IR), type 2 diabetes (T2D), metabolic syndrome (MS) and NAFLD [7,8,9]. However, the link between Fetuin-A and atherosclerotic CVD is more controversial. Some studies have suggested an association of CVD with either high or low Fetuin-A levels [10,11]. The different localizations of atherosclerosis may account for these conflicting results. 

Probably as a result of its virtually systemic nature, atherosclerosis is a heavily heterogeneous condition, which affects different age groups and has diverse risk factors and plaque features according to the arterial site [12,13,14,15,16]. For example, coronary artery disease (CAD) will typically anticipate the occurrence of peripheral arterial disease (PAD) by approximately ten years [17]. Moreover, coronary atherosclerosis is more frequently characterized by lipid core plaques; conversely, peripheral atherosclerosis is more typically fibro-calcific in its pathological nature [14,16].

To reconcile all the above discrepancies, we postulated that the interaction of NAFLD, Fetuin-A and atherosclerosis might vary based on the arterial site. Accordingly, this study aimed to explore the relationship of NAFLD and Fetuin-A with clinically relevant atherosclerosis affecting different localizations. Specifically, we assessed NAFLD prevalence, serum Fetuin-A values and their relationship with symptomatic CAD and PAD.

## 2. Patients and Methods

**Series**: Overall, the study population included 149 patients with established atherosclerotic CVD admitted either to the Cardiology Unit or Vascular Surgery Unit of Our Institution.

**Inclusion criteria**: Forty-five patients were consecutively recruited from the Cardiology Unit from April 2010 to July 2010 based on the results of an elective coronary angiography positive for CAD, defined as the presence of ≥50% stenosis in at least one main branch of coronary arteries. Coronary angiography was invariably indicated, in these patients, on the grounds for clinically suspected, symptomatic CAD. This group, already described in a previous work of our group [18], was named CAD group. Per clinical practice, individuals undergoing coronary angiography were not routinely submitted to procedures aimed at ruling out the presence of atherosclerosis at different localizations, unless clinically indicated.

One hundred and four patients, eligible for elective vascular surgery, were consecutively enrolled from the Vascular Surgery Unit from May 2013 to July 2014 based on the presence of carotid artery disease, lower extremity artery disease, or atherosclerotic thoracic and/or abdominal aortic aneurysm. In these patients, instrumental assessment of the peripheral arterial site was invariably indicated on the grounds of clinically suspected, symptomatic peripheral CVD. This group defined as PAD group consisted of: 23 symptomatic patients with Doppler ultrasound and computed tomography angiography imaging findings consistent with hemodynamically significant carotid artery stenosis; 55 symptomatic patients with lower extremity artery disease, confirmed by Doppler ultrasound and/or computed tomography angiography and/or lower extremity angiography; 26 patients with Doppler ultrasound and computed tomography angiography imaging findings consistent with atherosclerotic thoracic and/or abdominal aortic aneurysm. 

**Exclusion criteria** were: significant self-reported alcohol consumption (>30 g daily in men and >20 g daily in women); concurrent known aetiologies of chronic liver disease, notably viral, autoimmune, drug-induced and inherited (α1-antitrypsin deficiency, haemochromatosis or Wilson disease); either primary or metastatic liver cancer; end-stage renal disease on chronic renal replacement therapy. Competing etiologies of chronic liver disease were ruled out according to appropriate laboratory testing [19].

**Study protocol**: All recruited patients underwent an interview aimed at investigating their familial and personal history including concurrent diseases, metabolic co-morbidities, previous surgery and/or cardiovascular events, dietary habits, alcohol drinking and tobacco smoking, past and current use of medications, possible contacts with chemicals or drugs. During the hospitalisation period and before surgical procedures, physical examination, including measurement of anthropometric parameters and blood pressure, blood sampling and liver ultrasound were also performed.

Informed written consent was obtained from all participating individuals. The study was performed in agreement with the Declaration of Helsinki and the protocol of this study was approved by the local Ethical Committee (Prot. Num. 2159/C.E.).

**Laboratory tests**: Blood was sampled after an overnight fasting. Our metabolic laboratory evaluation included serum total cholesterol, high-density lipoprotein (HDL) cholesterol, triglycerides, fasting glucose and insulin. IR was calculated according to the homeostasis model assessment of IR (HOMA-IR) [20]; body mass index (BMI) ((kg)/height (m)^2^), T2D [21], arterial hypertension [22] and MS [23] were defined based on standard criteria. In all subjects, the presence of competing etiologies of chronic liver disease was ruled out by appropriate testing [19]. Finally, serum Fetuin-A was determined by a commercially available enzyme-linked immunosorbent assay (ELISA) (Fetuin-A (α2-HS Glycoprotein, AHSG) ELISA CE/IVD, 96 wells, BioVendor, Czech Republic).

**Ultrasound evaluation**: Liver ultrasound scanning was performed after overnight fasting, by an expert physician (either S.B. or D.R.), blinded to the biochemical and clinical data of patients, with a 3.5- to 5-MHz convex probe and a high-resolution B-mode scanner (Siemens Sonoline ANTARES^TM^, Erlangen, Germany). Fatty liver was diagnosed based on increased liver echogenicity compared to renal cortex. The severity of steatosis was evaluated according to the Ultrasonographic-Fatty Liver Indicator (US-FLI), a semiquantitative ultrasonographic score initially proposed and further developed by our group [24,25,26] and externally validated by others [27]. US-FLI ranges from 2 to 8. Diagnosis of steatosis was based on the presence of liver/kidney echo contrast, scored as mild/moderate (score 2) and severe (score 3). Additional criteria include the presence (score 1 each) or absence (score 0 each) of posterior attenuation of ultrasound beam, vessel blurring, difficult visualization of gallbladder wall or diaphragm, and areas of focal sparing [24,25,26]. The diagnosis of NAFLD was based on the presence of US-FLI ≥ 2 and exclusion of competing etiologies as detailed above [19]. 

**Statistical analysis**: Results were expressed as median (range, minimum−maximum) for continuous variables and as frequencies (percentages) for categorical variables. Comparisons between the medians of continuous variables were performed using the Mann–Whitney test. The Fisher exact test was used to compare nominal variables. Spearman’s Rho (ρ) was used to analyze correlations between Fetuin-A values and demographic, anthropometric, metabolic and ultrasonographic parameters. Two multivariate binary logistic regression analyses were performed to identify factors associated with NAFLD (vs. non-NAFLD) and CAD (vs. PAD). Odds ratios and 95% confidence intervals (CI) were reported with *p* values. Multivariate linear regression analysis was performed to identify factors associated with Fetuin-A values. Beta coefficients, standard errors (SE) and *p* values were reported. In order to avoid over-fitting of the multivariate analyses, we entered variables into the multivariate models based on: clinical relevance (i.e., age, NAFLD, T2D, Fetuin-A and/or CAD vs. PAD were invariably included into analyses) and on the results of univariate analyses (variables being chosen among those statistically significant at the univariate analysis). Moreover, in order to avoid co-linearity, we included, among the potential candidates, only those variables exploring physiologically distinct phenomena. Further to the overall study population, the analysis of factors associated with NAFLD and Fetuin-A was also separately performed in the CAD group and in the PAD group. A two-sided *p* value < 0.05 was considered to be significant. Statistical analyses were performed using the statistical software package SPSS, version 17.0 for Windows (SPSS Inc., Chicago, IL, USA).

## 3. Results

### 3.1. General Features of Study Population and Comparison of CAD vs. PAD Groups

In our series, approximately one in four individuals had multi-site artery disease based on medical history and even a larger proportion of patients may have had clinically silent multi-site artery disease. However, in this study, emphasis was given to the clinically relevant symptomatic arterial site at the time of enrolment. Indeed, the study protocol faithfully mirrored clinical practices and this justifies why not all different localizations of atherosclerosis were systematically evaluated. Furthermore, owing to the relatively small sample size we decided to analyze data collectively from a potentially heterogeneous group of patients who had peripheral atherosclerotic disease located at different arterial sites (carotid arteries, arteries of the lower extremities and aorta). We did so based on the preliminary finding that subgroup analysis had failed to identify significant differences (in term of demographic and metabolic variables, Fetuin-A levels and NAFLD prevalence) among patients with carotid artery disease versus lower extremity artery disease and atherosclerotic aortic aneurysm (data not shown).

Demographic, anthropometric and metabolic features of the overall study population, and according to CAD and PAD groups are shown in Table 1. 

Seventy-eight percent of patients were males; median age was of 72 (40–89) years. Metabolic comorbidities were common: median BMI was 27 (19–43) kg/m^2^, 36% of patients had T2D, 93% were hypertensive, 62% used lipid-lowering drugs and 50% had MS. NAFLD was detected in 54% of patients. Median Fetuin-A values were 256 (111–662) μg/mL. Patients with CAD were significantly younger, had lower HOMA-IR, less frequently had T2D and NAFLD (NAFLD prevalence 42% vs. 59%, *p* = 0.065; median (IQR) US-FLI 0 (0–3) vs. 2 (0–4), *p* = 0.038), but higher total cholesterol and Fetuin-A values (378 (124–662) vs. 236 (111–461), *p* < 0.001) than PAD patients.

### 3.2. Comparison of Non-NAFLD vs. NAFLD Patients

In the overall study population, NAFLD patients were significantly younger and had higher BMI, waist circumference, total cholesterol and triglycerides, and more frequently had T2D and MS than non-NAFLD patients. However, Fetuin-A values did not significantly differ between patients with NAFLD and their counterpart without NAFLD (268 (111–568) vs. 244 (124–662), *p* = 0.333) (Supplementary Table S1).

Comparison of non-NAFLD and NAFLD patients was also performed separately in the two groups of CAD and PAD patients. In the CAD group, NAFLD patients were significantly younger and with higher BMI, total cholesterol, triglycerides and Fetuin-A values than non-NAFLD patients. In the PAD group, patients with NAFLD still had higher BMI, waist circumference and total cholesterol, and suffered from T2D and MS more frequently than patients without NAFLD. However, no significant differences were found in age and Fetuin-A values between non-NAFLD and NAFLD patients in this group (Supplementary Table S1).

### 3.3. Correlations of Fetuin-A with Demographic, Anthropometric and Metabolic Parameters

In the overall group, Fetuin-A was weakly, though significantly and positively correlated with systolic and diastolic blood pressure, BMI, number of features of the MS, and total cholesterol. Moreover, there was a weak but significant inverse correlation of Fetuin-A with age. Indeed, patients older than 75 years (58 patients, 39% of the entire cohort) had significantly lower Fetuin-A values with respect to patients younger than 75 years (232 (111–537) vs. 273 (139–662), *p* = 0.011). No significant correlations were found with sex, HOMA-IR, creatinine, estimated glomerular filtration rate (eGFR) and US-FLI (Supplementary Table S2). Moreover, no differences in Fetuin-A values were found between patients with and without T2D (*p* = 0.279), and between patients with eGFR ≥ or < 60 mL/min/1.73 m^2^ (*p* = 0.250 for Cockroft–Gault equation).

In the CAD group, only US-FLI was found positively correlated with Fetuin-A, even if the association failed to reach full statistical significance (rho 0.261, *p* = 0.087). In the PAD group, Fetuin-A was weakly, though significantly and positively correlated with systolic blood pressure and number of MS features. No correlation was found between Fetuin-A and US-FLI in this group (rho 0.086, *p* = 0.390). (Supplementary Table S2).

**Figure 1:**
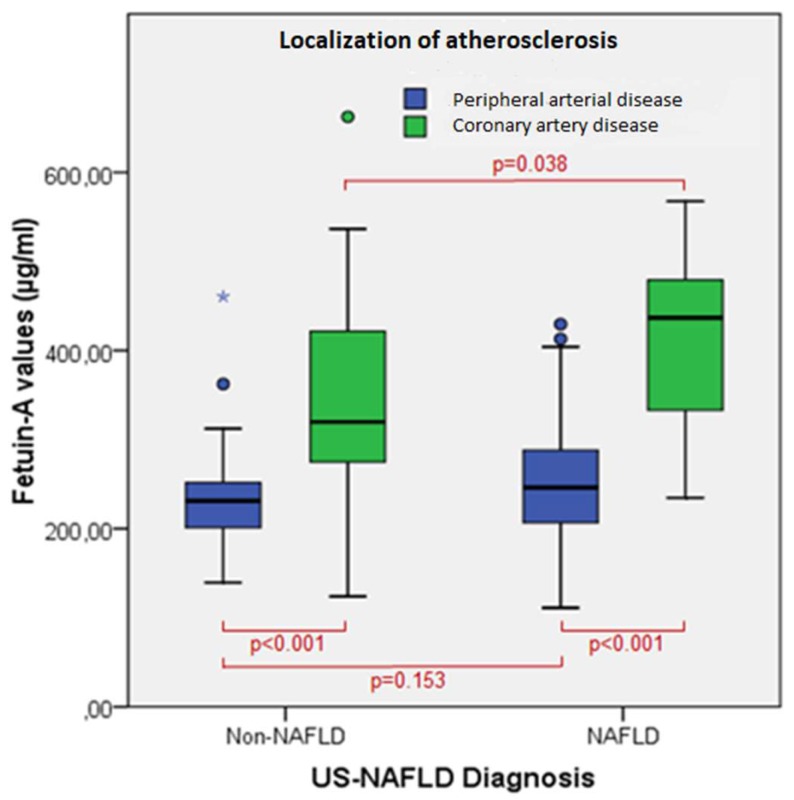
Fetuin-A values according to the presence of NAFLD and localization of atherosclerosis. Boxes represent the inter-quartile range and the line across the boxes indicates the median. The “whiskers” are the lines that extend from the box to the highest and lowest values, excluding outliers (dots and stars). The Mann–Whitney test was performed to compare medians. US = ultrasound.

Median Fetuin-A values were significantly higher in NAFLD than in non-NAFLD in CAD patients only. Moreover, the CAD group showed significantly higher values of Fetuin-A than the PAD group irrespective of the presence or absence of NAFLD (Figure 1) and irrespective of age. Indeed, the CAD group had significantly higher Fetuin-A values than the PAD group both in patients younger than 75 years (387 (139–662) vs. 239 (140–430), *p* < 0.001) and in patients older than 75 years (336 (124–537) vs. 221 (111–461), *p* = 0.009).

### 3.4. Factors Associated with CAD (vs. PAD), NAFLD and Fetuin-A at Multivariate Analyses

CAD (vs. PAD) was positively associated with Fetuin-A values (OR 1.02, 95% CI 1.01–1.02; *p* < 0.001) and negatively with NAFLD (OR 0.16, 95% CI 0.05–0.50; *p* = 0.002) (Table 2).

The factors associated with NAFLD in the overall study population were Fetuin-A (OR 1.01, 95% CI 1.00–1.01; *p* = 0.024), CAD group (OR 0.16, 95% CI 0.05–0.53; *p* = 0.003), BMI and T2D. In the CAD group, NAFLD was significantly associated with Fetuin-A (OR 1.01, 95% CI 1.00–1.02; *p* = 0.020) and BMI. In the PAD group, the factors positively associated with NAFLD were BMI and T2D, whereas Fetuin-A was not (Table 3).

In the overall study population, Fetuin-A was positively associated with CAD (Beta coefficient 131.36, SE 16.52; *p* < 0.001) and NAFLD (Beta coefficient 34.63, SE 16.44; *p* = 0.037). In the CAD group there was a trend towards a positive association between Fetuin-A and NAFLD independently of age, T2D and MS (Beta coefficient 77.77, SE 42.49; *p* = 0.075), which was not the case in the PAD group. (Table 4)

## 4. Discussion

Overall, the results of the present study are compatible with the postulate that the interaction among NAFLD, Fetuin-A and atherosclerosis probably varies based on the localization of atherosclerosis. More analytically, we have three main findings: (a) NAFLD is highly prevalent among all patients with definite atherosclerotic disease, though the highest prevalence rates are found among those patients with PAD; (b) despite a lower prevalence of NAFLD, patients with CAD have higher values of Fetuin-A than patients with PAD; (c) Fetuin-A is positively associated with NAFLD in CAD patients.

### 4.1. NAFLD Is Highly Prevalent among Patients with Definite Atherosclerotic Disease, though the Highest Prevalence Rates Are Found among Those Patients with PAD

More than 50% of patients in our series had NAFLD; in agreement with previous reports [28,29,30,31,32], this prevalence is significantly higher than that reported in the general population. Accordingly, further to dysmetabolic individuals [33], those with clinically significant atherosclerosis are another group at high risk for NAFLD. 

A novel finding in our study is that, compared to PAD individuals, CAD patients had a lower prevalence of NAFLD. A milder grade of IR and a lower prevalence of T2D in CAD patients may partly account for this finding. Of note, CAD patients were significantly younger than patients with PAD. It is tempting to speculate that the difference in NAFLD prevalence between CAD and PAD patients may also be explained by excess mortality (especially due to CAD) in NAFLD patients, as suggested by previous studies [34,35]. However, either selection bias or the limited number of recruited patients may have affected this finding, which prompts further investigation.

### 4.2. Patients with CAD Have Higher Values of Fetuin-A than Patients with PAD

Compared to PAD patients, CAD individuals had significantly higher Fetuin-A values. A dual role of Fetuin-A on cardiovascular biology and risk has been described. Studies have shown that (mainly among patients with end-stage renal failure) low Fetuin-A concentrations are associated with accelerated atherosclerosis and enhanced cardiovascular morbidity and mortality [11]. This finding may result from an impairment in the inhibitory role of circulating Fetuin-A on vascular calcification in vivo. Fetuin-A also enhances IR by inhibiting the intrinsic tyrosine kinase activity of the insulin receptor and, probably, by directly reducing adiponectin expression. Moreover, Fetuin-A seems to promote both adipose tissue inflammation and adipocyte dysfunction, by contributing to toll-like receptor 4 activation and macrophage migration and polarization into adipose tissue [36,37]. Finally, increased Fetuin-A levels are independently associated with T2D and MS development and correlate with the accumulation of hepatocyte triglycerides [7,8,9,38]. On these grounds, increasing evidence supports the notion that high, rather than low, Fetuin-A concentrations may be associated with atherosclerosis and cardiovascular events, especially in CAD [10]. However, studies investigating the relationship between Fetuin-A and atherosclerotic CVD are affected by high heterogeneity in patient populations, the different localizations of atherosclerosis, the presence/absence and stage of chronic kidney disease and T2D.

Our findings are compatible with the postulate that Fetuin-A may act differently depending on the specific arterial site affected by atherosclerosis. The finding of higher values of Fetuin-A observed in younger patients with CAD may suggest that this hepatokine plays a major role in promoting advanced atherosclerosis at the coronary site through IR. Conversely, the lower levels of Fetuin-A observed in older PAD patients may suggest that this hepatokine mainly exhibits impairment/failure of its anti-calcification activity at the peripheral arterial sites. Several lines of evidence support this view. Epidemiological studies have shown that CAD occurs in individuals who are approximatively ten years younger than those with peripheral atherosclerotic disease [17]. Moreover, imaging and pathological studies suggest that peripheral plaques tend to be more fibro-calcific than coronary plaques, which are more frequently lipid core plaques [14,16].

### 4.3. Fetuin-A Is Positively Associated with NAFLD in CAD Patients

NAFLD was independently associated with higher values of Fetuin A in our patients with clinically-relevant atherosclerotic CVD, even if this association seems to be limited to individuals affected by CAD. We cannot exclude that Fetuin-A levels in CAD patients may reflect MS rather than NAFLD. However, a statistical trend toward an association of fetuin-A with NAFLD was found independently of T2D and MS.

Fetuin-A is mainly synthesized by hepatocytes, hence liver dysfunction may affect serum Fetuin-A concentration. Increasing evidence supports that liver steatosis stimulates the synthesis of Fetuin-A [5,6,7]. Accordingly, serum Fetuin-A levels increase in patients with NAFLD proportionally to hepatic triglyceride content and, conversely, they are reduced by body weight loss, aerobic excercise and reduction of the grade of steatosis [39,40]. Moreover, Fetuin-A has been associated with inflammation and endothelial dysfunction; specifically, it has been shown that circulating Fetuin-A levels are independently associated with endothelial dysfunction and subclinical atherosclerosis in patients with NAFLD [41], suggesting that this hepatokine may be an important mediator of the atherogenic potential of NAFLD.

### 4.4. Limitations and Strengths

We acknowledge that our study has several limitations. Firstly, the study population appears relatively low; this may account for the mainly weak correlations we found in the study and, for the same reason the results of multivariate analysis should be interpreted with caution. Secondly, the groups are very co-morbid, quite heterogeneous in term of age and renal function, and not closely balanced given that this study faithfully reflects clinical practice. In the attempt to overcome the heterogeneity in age we have performed sensitivity analyses by stratifying the groups according to age and we have included age as a covariate in all the analyses. It is a matter of debate whether Fetuin-A concentrations are directly related to renal function. Lower Fetuin-A concentrations have consistently been reported in patients with end-stage renal disease on dialysis [11]. To overcome the potential confounding effect of renal function, we excluded patients on chronic renal replacement therapy; moreover, only a minority of our patients had severe chronic kidney disease assessed according to eGFR. Previous studies failed to demonstrate that mild-to-moderate chronic kidney disease is associated with lower concentrations of Fetuin-A [42]. Consistently, we did not find any correlation between eGFR and Fetuin-A values in our cohort. Thirdly, owing to its cross-sectional design, our analysis describes associations but does not prove a causal link between NAFLD, Fetuin-A and atherosclerosis. Fourthly, age- and sex-matched healthy controls and NAFLD patients without CVD were not recruited in this study, given that we wanted to compare CAD vs. PAD patients. Moreover, owing to the virtually systemic nature of atherosclerosis, a proportion of patients may have had clinically silent multi-site artery disease. Nevertheless, we also emphasize that the rationale for dividing the cohort into CAD and PAD is strongly based on those specific clinical manifestations of often severely symptomatic individual vascular patients: these specific clinical manifestations were the main and most relevant focus of our research. The study protocol followed here has tried to faithfully mirror clinical practices and this accounts for not all PAD patients undergoing coronary angiography. Similarly, not all CAD-patients were submitted to systematic evaluation of other localizations of atherosclerosis. Additionally, we acknowledge that the two groups of CAD and PAD patients were recruited at different times, three years apart. However, the same study protocol and the same methods of measurements (notably including Fetuin-A assay and ultrasonographic fatty liver score) were applied for each group, irrespective of the time of recruitment. On these grounds, we strongly believe that parameters are reliably comparable. Finally, although some would argue that ultrasonography is relatively inaccurate in diagnosing and excluding NAFLD, it does remain the first-line imaging technique in diagnosing NAFLD both in clinical practice and in epidemiological studies. Of note, the semi-quantitative ultrasonographic score used in this study, US-FLI, has been externally validated [27] and has recently been reported to be able to detect as little steatosis as 10% in our hands [26].

Studies have addressed the link between Fetuin-A and CVD or Fetuin-A and MS/NAFLD. However, we emphasize that ours is a comparative study specifically aimed at exploring the relationship of NAFLD and Fetuin-A with clinically relevant symptomatic atherosclerosis affecting different arterial sites.

## 5. Conclusions

Our findings are fully consistent with previous studies [5,6,7] and, for the first time, extend the positive association of Fetuin-A with NAFLD also to a group of patients with established atherosclerotic CVD. However, when subgroup analyses were performed based on the arterial site affected by atherosclerosis, NAFLD was found to be associated with higher Fetuin-A values in CAD patients alone. This finding supports the postulate that the association of Fetuin-A, NAFLD and atherosclerosis varies depending on the localization of atherosclerosis. We speculate that Fetuin-A could modulate the effect of age by exhibiting elevated values, strongly associated with NAFLD, in CAD, which is more prone to lipid core plaques, and decreased values, weakly associated with NAFLD, in PAD, more commonly characterized by fibro-calcific plaques. This hypothesis should be either confirmed or rejected by larger prospective studies.

## Figures and Tables

**Table 1:** Clinical features of study population and comparison of coronary artery disease vs. peripheral arterial disease groups.

Parameter	Overall Study Population (*n* = 149)	Coronary Artery Disease Group (*n* =4 5)	Peripheral Arterial Disease Group (*n* = 104)	*p*
Age (years)	72 (40–89)	69 (40–89)	73 (53–89)	0.040
Male sex (n (%))	116 (78)	34 (76)	82 (79)	0.657
BMI (Kg/m^2^)	27 (19–43)	27 (19–43)	26 (20–35)	0.211
Waist circumference (cm)	101 (70–133)	100 (70–132)	102 (76–133)	0.447
Type 2 diabetes mellitus (n (%))	53 (36)	10 (22)	43 (41)	0.025
Arterial hypertension (n (%))	138 (93)	39 (87)	99 (95)	0.068
Lipid lowering drugs (n (%))	92 (62)	31 (69)	61 (59)	0.265
Metabolic syndrome (n (%))	74 (50)	19 (42)	55 (53)	0.232
Smoking status (n (%)) Non-smokers Ex-smokers Active smokers	30 (20) 62 (42) 57 (38)	15 (33) 1 (2) 29 (64)	15 (14) 61 (59) 28 (27)	<0.001
US-FLI	2 (0–8)	0 (0–8)	2 (0–8)	0.038
NAFLD (US-FLI ≥ 2) (n (%))	80 (54)	19 (42)	61 (59)	0.065
Glucose (mg/dL)	103 (44–275)	98 (64–197)	108 (44–275)	0.181
HOMA-IR (mg/dL × μIU/mL)	1.6 (0.2–18.5)	1.3 (0.3–7.3)	1.8 (0.2–18.5)	0.009
Total cholesterol (mg/dL)	161 (68–296)	175 (123–280)	156 (68–296)	0.023
HDL cholesterol (mg/dL)	40 (16–90)	41 (16–77)	40 (20–90)	0.988
Triglycerides (mg/dL)	118 (37–451)	116 (37–287)	118 (57–451)	0.291
ALT (U/l)	17 (1–268)	23 (7–268)	14 (1–74)	<0.001
AST (U/l)	19 (6–395)	21 (12–395)	19 (6–80)	0.020
GGT (U/l)	30 (8–348)	27 (13–105)	34 (8–348)	0.259
Alkaline phospatase (U/l)	77 (36–502)	70 (36–502)	82 (38–257)	0.003
Creatinine (mg/dL)	1.0 (0.4–11.1)	0.9 (0.6–3.0)	1.0 (0.4–11.1)	0.420
eGFR (mL/min/1.73 m^2^) Cockroft–Gault equation eGFR ≥ 60 eGFR ≥ 30 and < 60 eGFR < 30	72 (4–170) 97 (66) 38 (26) 13 (9)	79 (18–170) 34 (76) 7 (16) 4 (9)	67 (4–145) 63 (61) 31 (30) 9 (9)	0.070 0.168
Platelets (1000/mL)	198 (89–668)	182 (104–320)	207 (89–668)	0.084
Fetuin-A values (µg/mL)	256 (111–662)	378 (124–662)	236 (111–461)	<0.001

Data were expressed as median (range, minimum–maximum) for continuous variables and as frequencies (percentages) for categorical variables. BMI: body mass index; US-FLI: ultrasonographic-fatty liver indicator; NAFLD: nonalcoholic fatty liver disease; HOMA-IR: homeostasis model assessment of insulin resistance; eGFR: estimated glomerular filtration rate.

**Table 2:** Multivariate analysis exploring factors associated with coronary artery disease (vs. peripheral arterial disease).

Parameter	OR (95% CI)	*p*
Fetuin-A *	1.02 (1.01–1.02)	<0.001
NAFLD (US-FLI ≥ 2) **	0.16 (0.05–0.50)	0.002
Age *	0.96 (0.92–1.01)	0.154
Type 2 diabetes mellitus **	0.83 (0.28–2.40)	0.723
Total cholesterol *	1.01 (1.00–1.02)	0.111

Model including Fetuin-A, NAFLD, age, type 2 diabetes mellitus and total cholesterol. *per unit; ** yes vs. no. NAFLD: nonalcoholic fatty liver disease; US-FLI: ultrasonographic-fatty liver indicator; HOMA-IR: homeostasis model assessment of insulin resistance. OR: odds ratio; CI: confidence interval.

**Table 3:** Multivariate analysis exploring factors associated with NAFLD.

Parameter	Overall Study Population		Coronary Artery Disease Group		Peripheral Arterial Disease Group	
	OR (95% CI)	*p*	OR (95% CI)	*p*	OR (95% CI)	*p*
Fetuin-A *	1.01 (1.00–1.01)	0.024	1.01 (1.00–1.02)	0.020	1.00 (1.00–1.01)	0.353
Coronary artery disease **^§^	0.16 (0.05–0.53)	0.003	-	-	-	-
Age *	0.96 (0.92–1.00)	0.067	0.94 (0.88–1.01)	0.100	0.97 (0.92–1.03)	0.294
BMI *	1.18 (1.06–1.31)	0.003	1.40 (1.05–1.88)	0.024	1.14 (1.00–1.29)	0.045
Type 2 diabetes mellitus **	3.40 (1.49–7.75)	0.004	5.32 (0.75–37.75)	0.094	3.09 (1.23–7.78)	0.017

Model for the overall study population including Fetuin-A, coronary artery disease, age, BMI and type 2 diabetes mellitus; model for coronary artery disease group including Fetuin-A, age, type 2 diabetes mellitus and BMI; model for peripheral arterial disease including Fetuin-A, age, BMI and type 2 diabetes mellitus. * per unit; ** yes vs. no; ^§^ vs. peripheral arterial disease. NAFLD: nonalcoholic fatty liver disease; BMI: body mass index. OR: odds ratio; CI: confidence interval.

**Table 4:** Multivariate analysis exploring factors associated with Fetuin-A.

Parameter	Overall Study Population			Coronary Artery Disease Group			Peripheral Arterial Disease Group		
	Beta Coefficient	SE	*p*	Beta Coefficient	SE	*p*	Beta Coefficient	SE	*p*
Coronary artery disease **^§^	131.36	16.52	<0.001	-	-	-	-	-	-
NAFLD (US-FLI ≥ 2) **	34.63	16.44	0.037	77.77	42.49	0.075	10.41	12.47	0.406
Age *	−0.690	0.801	0.390	−0.074	1.747	0.967	−0.844	0.702	0.232
BMI *	0.059	1.939	0.976	-	-	-	-	-	-
Total cholesterol *	−0.005	0.184	0.977	-	-	-	-	-	-
Type 2 diabetes mellitus **	−16.43	16.05	0.308	−25.88	48.75	0.598	−32.80	13.07	0.014
Metabolic syndrome **	-	-	-	6.452	40.04	0.873	52.33	12.66	<0.001

Model for the overall study population including coronary artery disease, NAFLD, age, BMI, total cholesterol and type 2 diabetes mellitus; model for coronary artery disease group including NAFLD, age, type 2 diabetes mellitus and metabolic syndrome; model for peripheral arterial disease including NAFLD, age, type 2 diabetes mellitus and metabolic syndrome. * per unit; ** yes vs. no; ^§^ vs. peripheral arterial disease. NAFLD: nonalcoholic fatty liver disease; US-FLI: ultrasonographic-fatty liver indicator; BMI: body mass index.

## Supplementary Materials

The following are available online. Table S1: Comparison of non-NAFLD vs. NAFLD patients; Table S2: Correlations of Fetuin-A with demographic, anthropometric and metabolic parameters.

Supplementary File 1

## Abbreviations

BMI	body mass index
CAD	coronary artery disease
CI	confidence interval
CVD	cardiovascular disease
eGFR	estimated glomerular filtration rate
HDL	high-density lipoprotein
HOMA-IR	homeostasis model assessment of insulin resistance
IR	insulin resistance
MS	metabolic syndrome
NAFLD	nonalcoholic fatty liver disease
OR	odds ratio
PAD	peripheral arterial disease
SE	standard error
T2D	type 2 diabetes
US-FLI	ultrasonographic-fatty liver indicator
