# Supplementary material for: Do Nonalcoholic Fatty Liver Disease and Fetuin-A Play Different Roles in Symptomatic Coronary Artery Disease and Peripheral Arterial Disease?
Source: Diseases. 2018 Feb 16;6(1):17. doi: 10.3390/diseases6010017 (PMC5871963; doi:10.3390/diseases6010017)
Supplement: Supplementary File 1 [file diseases-06-00017-s001.docx]

| **Supplementary Table 1. Comparison of non-NAFLD vs. NAFLD patients** | | | | | | | | | |
| --- | --- | --- | --- | --- | --- | --- | --- | --- | --- |
|  | **Overall study population** | | | **Coronary artery disease group** | | | **Peripheral arterial diseases group** | | |
|  | **Non-NAFLD (n=69)** | **NAFLD (n=80)** | **p** | **Non-NAFLD (n=26)** | **NAFLD (n=19)** | **p** | **Non-NAFLD (n=43)** | **NAFLD (n=61)** | **p** |
| **Age (years)** | 73 [40-89] | 70 [45-87] | 0.037 | 73 [40-89] | 68 [45-78] | 0.018 | 73 [55-89] | 72 [53-87] | 0.181 |
| **Male sex [n (%)]** | 53 (77) | 63 (79) | 0.776 | 20 (77) | 14 (74) | 0.803 | 33 (77) | 49 (80) | 0.659 |
| **BMI (Kg/m^2^)** | 25 [19-34] | 28 [20-43] | <0.001 | 26 [19-33] | 28 [20-43] | 0.004 | 25 [20-34] | 27 [20-35] | 0.002 |
| **Waist (cm)** | 98 [70-129] | 104 [80-133] | <0.001 | 98 [70-114] | 104 [80-132] | 0.052 | 97 [76-129] | 104 [83-133] | 0.004 |
| **Type 2 diabetes mellitus [n (%)]** | 14 (20) | 39 (49) | <0.001 | 3 (12) | 7 (37) | 0.070 | 11 (26) | 32 (53) | 0.006 |
| **Arterial hypertension [n (%)]** | 64 (93) | 74 (93) | 0.953 | 23 (89) | 16 (84) | 0.679 | 41 (95) | 58 (95) | 0.950 |
| **Lipid lowering drugs [n (%)]** | 39 (57) | 53 (66) | 0.266 | 16 (62) | 15 (79) | 0.213 | 23 (55) | 38 (62) | 0.445 |
| **Metabolic syndrome [n (%)]** | 26 (38) | 48 (60) | 0.007 | 9 (35) | 10 (53) | 0.227 | 17 (40) | 38 (62) | 0.022 |
| **HOMA-IR (%)** | 1.4 [0.2-11.4] | 1.8 [0.2-18.5] | 0.159 | 1.1 [0.5-4.5] | 1.5 [0.3-7.3] | 0.267 | 1.8 [0.2-11.4] | 1.8 [0.2-18.5] | 0.737 |
| **Total cholesterol (mg/dl)** | 153 [68-256] | 171 [76-296] | 0.004 | 163 [123-198] | 182 [128-280] | 0.002 | 148 [68-256] | 167 [76-296] | 0.029 |
| **HDL cholesterol (mg/dl)** | 40 [20-90] | 40 [16-69] | 0.796 | 39 [23-77] | 42 [16-69] | 0.774 | 40 [20-90] | 39 [26-67] | 0.896 |
| **Triglycerides (mg/dl)** | 108 [37-246] | 129 [57-451] | 0.007 | 91 [37-246] | 145 [69-287] | 0.018 | 114 [57-246] | 121 [57-451] | 0.170 |
| **Fetuin-A values (µg/ml)** | 244 [124-662] | 268 [111-568] | 0.333 | 320 [124-662] | 437 [254-568] | 0.038 | 231 [140-461] | 246 [111-430] | 0.153 |

Data were expressed as median [range, minimum-maximum] for continuous variables and as frequencies (percentages) for categorical variables.

NAFLD: non-alcoholic fatty liver disease; BMI: Body Mass Index; HOMA-IR: homeostasis model assessment of insulin resistance; ALT: alanine aminotransferase.

| **Supplementary Table 2. Correlations of Fetuin-A with demographic, anthropometric and metabolic parameters** | | | | | | |
| --- | --- | --- | --- | --- | --- | --- |
|  | **Overall study population** | | **Coronary artery disease group** | | **Peripheral arterial diseases group** | |
|  | **Spearman’s rho** | **p** | **Spearman’s rho** | **p** | **Spearman’s rho** | **p** |
| **Age** | - 0.250 | 0.002 | - 0.132 | 0.391 | - 0.162 | 0.104 |
| **Systolic pressure** | 0.213 | 0.010 | - 0.123 | 0.425 | 0.329 | 0.001 |
| **Diastolic pressure** | 0.204 | 0.014 | - 0.115 | 0.458 | 0.105 | 0.292 |
| **BMI** | 0.207 | 0.012 | 0.022 | 0.886 | 0.183 | 0.066 |
| **Waist** | 0.012 | 0.887 | - 0.159 | 0.307 | 0.032 | 0.749 |
| **N° of Metabolic Syndrome Features** | 0.215 | 0.009 | 0.037 | 0.813 | 0.318 | 0.001 |
| **Total cholesterol** | 0.177 | 0.032 | - 0.069 | 0.658 | 0.184 | 0.064 |
| **HDL cholesterol** | 0.020 | 0.814 | 0.018 | 0.910 | 0.095 | 0.345 |
| **Triglycerides** | 0.077 | 0.357 | 0.089 | 0.567 | 0.121 | 0.230 |
| **HOMA-IR** | -0.023 | 0.786 | - 0.066 | 0.672 | 0.151 | 0.131 |
| **Creatinine** | 0.069 | 0.406 | 0.080 | 0.605 | 0.164 | 0.100 |
| **eGFR MDRD equation**  **eGFR Cockroft-Gault equation** | -0.033  0.140 | 0.693  0.092 | -0.002  0.040 | 0.988  0.799 | -0.150  -0.007 | 0.133  0.942 |
| **US-FLI** | 0.022 | 0.795 | 0.261 | 0.087 | 0.086 | 0.390 |

BMI: Body Mass Index; HDL: high-density lipoprotein; HOMA-IR: homeostasis model assessment of insulin resistance; ALT: alanine aminotransferase; US-FLI: Ultrasonographic-Fatty Liver Indicator; eGFR: estimated glomerular filtration rate; MDRD: Modification of Diet in Renal Disease
